# Wild Animals Used as Food Medicine in Brazil

**DOI:** 10.1155/2013/670352

**Published:** 2013-08-06

**Authors:** Rômulo Romeu Nóbrega Alves, Tacyana Pereira Ribeiro Oliveira, Ierecê Lucena Rosa

**Affiliations:** ^1^Departamento de Biologia, Universidade Estadual da Paraíba, Avenida das Baraúnas, 351/Campus Universitário, Bodocongó 58109-753, Campina Grande, PB, Brazil; ^2^Centro de Ciências Biológicas e Sociais Aplicadas, Universidade Estadual da Paraíba, Rua Horácio Trajano de Oliveira, s/n, Campus V, 58020-540 João Pessoa, PB, Brazil; ^3^Departamento de Sistemática e Ecologia, Universidade Federal da Paraíba, 58059-900 João Pessoa, PB, Brazil

## Abstract

The connection between eating and healing is common in traditional folk medical systems, and the multiple possibilities resulting from the combination of biodiversity and culture confer a wealth and complexity in terms of knowledge of the flora and fauna as to their potential as food medicine. The growing awareness of the links between traditional therapeutic-alimentary uses of wildlife and conservation has drawn attention to the gaps in knowledge on the social, economic, and biological contexts in which different forms of traditional wildlife uses take place, particularly with regard to zootherapeutic resources. In this study we interviewed 124 merchants and 203 traditional users of animal-derived remedies in Brazil, aiming at documenting the animal species used as foods and medicines in urban and rural areas of the country. At least 354 wild animal species are used in Brazilian traditional medicine, of which 157 are also used as food. The high degree of overlap between medicinal and alimentary uses of wild animals highlights the importance of understanding the socioeconomic, cultural, and ecological contexts in which those traditional uses take place for elucidating their potential impact on public health and biodiversity conservation.

## 1. Introduction

Nature-based traditional food and medicine are generally viewed as interchangeable, diet being highly regarded as the primary basis for sustaining and/or restoring health and well-being. Consequently, foods are considered and often times chosen for their distinctive medicinal or healing values [1–4]. For many traditional societies, now and in the past, food is—at least partially—medicine, and medicine is food [5]. Medicinal cuisines and consumption of health edibles have held a central position in traditional folk medical systems [6–10].

Much of the plant material that is consumed by animals in the wild contains an array of secondary compounds. Johns [6] argues that the herbal medicines and modern pharmaceuticals used by humans today have replaced the nonnutritive chemicals commonly present in our primate ancestors' diets, the connection between eating and healing likely being as old as the use of biodiversity by humans [6]. For ancient Assyrians, Greeks, and Chinese, cultivated grains, roots, fruits, and spices were frequently employed in the curing arts, while, in pharaonic Egypt, plants such as lettuce, sesame, onions, leeks, cucumbers, plums, watermelon, and many other edibles were included in the healer's arsenal [11]. Historical sources of ancient Egypt also mention the medicinal uses of animal-derived substances, such as cattle milk, bee honey, lizard blood, ox organs, swallow's liver, bat limbs, ambergris from the sperm whale, and the glands of the musk deer [12–15].

At the dawn of recorded history it is known that man often ate or wore on his person some portion of an animal that was thought to have a healing or protecting influence [16], and this aspect highlights that the origin of the medicinal use of faunal elements is intertwined with their use as food. In this same direction, Chemas [17] remarked that the treatment of illnesses using animal-based remedies is an extremely old practice, whose most remote ancestor is a carnivore diet, closely followed by the ritual ingestion of deceased persons (e.g., close relatives, warriors) as a means to absorb their virtues (e.g., courage, virility) and subsequently by a true medicinal use indissociable from magic-religious elements.

Since ancient times, the thinking of “food as medicine” has existed in Chinese medical theories and Chinese food therapy [18, 19]. Books on Chinese cooking often emphasize the medicinal value of foods and the importance of “nutritional therapies” dating from earliest times, and many of the vegetable and animal products decocted in Chinese medicines are used routinely in cooking. During the 1980s, talk at banquets frequently revolved around the healthful properties of foods being consumed, and nutritional and food preparation advice was commonly tendered in clinics along with herbal prescriptions [20].

Research in several regions of the world has illustrated that many wild plants retained in local food cultures are inseparable from traditional therapeutic systems [7, 9, 21, 22]. For example, in a rural Hausa community in northern Nigeria, of the 119 plants identified as food, all but five are included among the total 374 medicinals. This, however, does not mean that Hausa intermixes the domains of food and medicine [7]. Examples of a number of food animals also used as remedies can be found in the literature [23–28]. Yet our knowledge about the practice of food medicine is limited, particularly with regard to the traditional consumption of animal food medicines [29].

Although often regarded as supplementary to local peoples' diet, wild food and medicine are essential in times of crisis and play an important nutritional role. Hence, the neglect of traditional food and medicines may seriously deteriorate the health and well-being of traditional peoples [30, 31]. Further, nature-based traditional food and medicine are generally viewed as interchangeable, diet being highly regarded as the primary basis for sustaining and/or restoring health and well-being. Consequently, foods are considered and often times chosen for their distinctive medicinal or healing values.

Animal medicinal foods have been broadly used since ancient times and have played a significant role in healing practices in Brazil [3, 32], where elements of indigenous, European, and African cultures met and produced a singular repertoire of species that are used as food and often also as medicine.

Brazil provides an interesting setting for several reasons: (a) the country possesses between 15 and 20% of all the world's biological diversity, as well as a significant cultural diversity, represented by more than 200 indigenous groups and by a large number of local communities which detain a considerable knowledge of the flora and fauna and of traditional systems of renewable natural resources management [25]; (b) the multiple possibilities resulting from this combination of biodiversity and culture confer a wealth and complexity in terms of knowledge of the Brazilian flora and fauna as to its therapeutic potential; (c) in addition, Brazil is vast, with parts of the territory of difficult access; this precludes some local populations from accessing services provided by the government's health care network. In many cases this geographical isolation contributes to strengthen traditional and local medical practices and, also, to prompt selection of natural resources for the treatment of new diseases [25, 33]. Interest in animal-derived remedies, however, extends beyond people lacking access to medical services in Brazil. As shown by Alves and Rosa [26, 27], even in cities where such services are more accessible, many people still resort to traditional healers, showing the cultural acceptability of such practices.

In this study we explored the medicinal and alimentary uses of wildlife in Brazil, aiming to (1) document the animal species used and the illnesses to which they were prescribed and (2) to discuss resource use in a conservationist context.

## 2. Methods

Data were collected from January 2002 to June 2012. Data consisted in (a) interviews in markets/shops located in the cities of Belém (Pará State), São Luís (Maranhão State), Teresina (Piauí State), Goiânia (Goiás State), Natal (Rio Grande do Norte State), João Pessoa, Campina Grande (Paraíba State), Recife (Pernambuco State), Maceió (Alagoas State), Aracajú (Sergipe State), Salvador (Bahia State), Vitória (Espírito Santo State), Niterói (Rio de Janeiro State), Florianópolis (Santa Catarina State), and Porto Alegre (Rio Grande do Sul State), where we documented the animal medicinal foods traded, (b) interviews in outdoor markets to 124 merchants about the use and commercialization of medicinal animals (23 interviewees in Belém, 21 in São Luís, 21 in Teresina, 16 in Santa Cruz, 11 in Caruaru, 10 in João Pessoa, and 22 in Campina Grande), (c), and interviews with 203 traditional users of animal-derived remedies (67 men and 70 women) in the following rural communities: Municipality of Cajueiro da Praia (Piauí State) (*n* = 36), Pesqueiro Beach, Municipality of Soure (Pará State) (*n* = 41), Environmental Protected Area Barra do Rio Mamanguape, Municipality of Rio Tinto (Paraíba State) (*n* = 30), Municipality of Queimadas (Paraíba State) (*n* = 66), and Municipality of Raposa (Maranhão State) (*n* = 30), as described in Alves and Rosa [1, 25, 26].

In cities, the sampling method was nonrandom, and the interviewees were predefined [34]. Despite attempts to interview all animal merchants in the markets visited, some interviews were cancelled. Others proved to be fruitless, because interviewees were reluctant to answer questions. At the surveyed fishing communities, we identified local people with a specialized knowledge of medicinal animal use. Additional interviewees were chosen by using the snowball technique [35], based on information initially provided by the specialists.

To respect intellectual property rights, we adopted the following protocol in the field: before the survey, we introduced ourselves, explained the nature and objectives of our research, and asked the respondents for permission to record the information. The ethical approval for the study was obtained from the Ethics Committee of Paraiba University State.

The information obtained through semistructured interviews was complemented by free interviews [36], and, for each animal cited, respondents were requested to furnish vernacular name, folk use, parts used, preparation and administration of remedy, and which animal species are also used as food. Zoological material was identified with the aid of specialists, through (1) examination of voucher specimens (donated by the interviewees) (2) photographs of the animals or their parts, taken during interviews; and (3) vernacular names, with the aid of taxonomists familiar with the study areas' fauna. Only wild animals and taxa that could be identified to species level were included in the database.

Records of animal-based folk remedies were gathered from scientific articles, books, and book chapters, theses, and dissertations, as well as from reports available in international online databases such as Science Direct (http://www.sciencedirect.com/), Scirus (http://www.scirus.com/), Google Scholar, Scopus (http://www.scopus.com/), Web of Science (http://www.isiknowledge.com/), and Biological Abstracts (http://science.thomsonreuters.com/) using the following search terms: medicinal animals + use + Brazil, zootherapy + commercialization + Brazil, hunting + medicinal animals + Brazil, and, and zootherapy + Brazil.

Whenever applicable, scientific names provided in publications were updated using ITIS Catalogue of Life: 2012 Annual Checklist (http://www.catalogueoflife.org/).

Information on the conservation status of animal species was obtained from the International Union for Conservation of Nature's Red List (http://www.iucnredlist.org/), the Convention on the International Trade in Endangered Species of Wild Fauna and Flora (http://www.cites.org/eng/resources/species.html), Brazil's Official List of Endangered Species [37] and National List of Species of Aquatic Invertebrates and Fishes Endangered, Overexploited, or Threatened of Exploitation [38].

## 3. Results and Discussion

**Figure 1:**
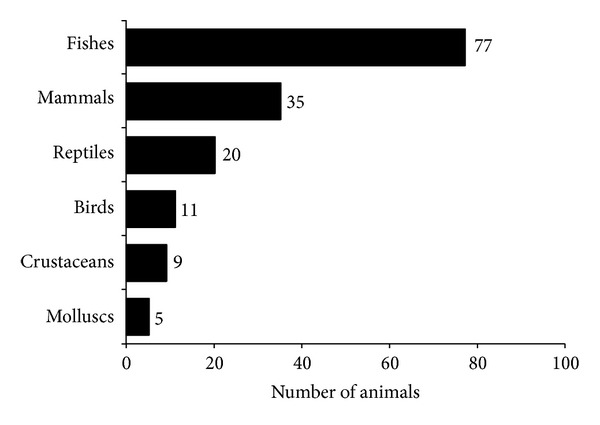
Number of animal species, per taxonomic category, used as food and medicines in Brazil.

Animals used as medicine food recorded in our study were distributed in six zoological groups. As shown in Figure 1, the taxon with the largest number of species was fishes (77 species; 49.0%), followed by mammals (35; 22.3%) and reptiles (20; 12.7%). These results are in line with previous studies carried out elsewhere (eg., 28, 40, and 41–46), further highlighting the widespread use of wild-caught vertebrates in the diets and medical systems of different societies.

Generally, species are harvested through fishing or hunting, mainly for alimentary purposes. In this sense, their utilization as remedies potentializes resource use. Meat, the principal product consumed as food, in some cases was also consumed due to a perceived medicinal value. This distinction, however, was not always clear-cut, as the same animal can be one, the other, or both categories at the same time, depending on the parts used, the method of preparation, and the state of health or pathology of the individual being treated. This result is in line with Huffman [39], who remarked that in traditional human societies, the difference between food and medicine may not always be clear. In fact, according to O'Hara-May [40], the beginnings of the medicinal uses of animals in human history are clear: animals and their products were part of the primary resources that ancient peoples could use as food or for treating their illnesses.

Most of the time, the hunted or fished animal whose meat is consumed as food also provides byproducts that are used for medicinal purposes, such as skin or fats. In fact, the utilization of remainings or by-products seems to be widespread and one of the most striking characteristics of the Brazilian folk medicine, in terms of medicinal animals [41]. In this sense, it is remarkable that in general the animal-based medicinal products constitute by-products from animals hunted for other purposes. Such multiple uses (including medicinal) of fauna and their impact on animal populations must be properly assessed and taken into consideration when implementing recovery plans for these target species, especially those that are highly exploited [3, 27, 42].

The high number of fish species recorded as medicinal foods in this study was expected, given their high consumption as food, mainly in coastal areas. As pointed by Burger and Gochfeld [43], in many parts of the world more than half of the people live in coastal communities where fish is prominent in their diets. Although people who live near the sea eat more seafood than those who live in the hinterland, seafood, both fresh and frozen, has become increasingly available and is gaining in popularity throughout the world. In addition to being an important and available source of protein, the popularity of fish as food is also due to the fact that they are considered healthy. Fishes are considered an excellent and low-fat source of protein, provide many health benefits, such as omega-3 fatty acids that reduce cholesterol levels and the incidence of stroke, heart disease, and preterm delivery, and enhance cognitive development [44–52].

Among terrestrial vertebrates, mammals are the most hunted taxon for alimentary purposes. The frequent use of those animals as medicinal foods was expected, given their comparatively larger body size (when compared with other terrestrial vertebrates) and the possibility they offer of a higher energetic intake. In the neotropical region, mammals clearly constitute the most important taxonomic group in terms of the number of species used by rural communities [53–60].

Reptiles ranked third in the number of species recorded used as food and medicine in this study and are among the animals most frequently used in folk medicine; the consumption of reptile meat is often intertwined with cultural or medicinal beliefs [61–67]. In this study, chelonians stood out as the reptiles most used as medicinal food (*n* = 13 species), a result in line with their extensive use as food in Brazil. As shown by Alves et al. [66], of the 36 species of chelonians in Brazil, 20 (55.5%) are eaten by humans. Those animals are commonly sought after as food in the northern region of the country where they achieve the highest species richness and abundance. In a smaller proportion, lizards and caymans are also important as food medicine; on the other hand, only a few snake species have been used as food, despite the reported use of several species in Brazilian traditional medicine [64, 66]. The small number of snake species currently used as food in Brazil is not surprising given the negative images attributed to these animals in myths, legends, and popular beliefs [66–68]. Rea [69] noted that not only snakes are rejected because of their disagreeable nature, but also any other creature with a similar shape or behavior will receive similar treatment. A study undertaken among human populations living along the banks of the Rio Negro River (Amazonas State, Brazil) showed that the electric eel (*Electrophorus electricus*) was one of the least favored meats because of its strong smell and the shape of its body—“it looks just like a snake” [70].

Some of the animals quoted by interviewees were mainly hunted for medicinal purposes, an example being the boa snake (*Boa constrictor*), which is eventually also used as food [66]. Conversely, other species are hunted for consumption, and their byproducts are utilized for medicinal purposes. At least 354 wild animal species are used in Brazilian traditional medicine [71], of which 157 (44.3%) are also used as food (Table 1), a result that mirrors the central role played by wildlife as a source of protein in different parts of the world. As shown by previous studies, in at least 62 countries worldwide, wildlife (including fish) provides significant proteins, calories, and essential fats to rural communities [58–60, 72–76]. It should be noted, however, that the number of animal species used as medicinal food in Brazil was higher than the number of species recorded for those purposes elsewhere (see [23]), possibly as a result of the country's significant biological and cultural diversity [77].

The overlap between alimentary and medicinal use can be exemplified by the use of caymans (*Caiman latirostris, C. crocodilus, Paleosuchus palpebrosus,* and *Melanosuchus niger*). While their meat was primarily consumed as food, their teeth, skin, fat, and penis were used for treating diseases such as asthma, stroke, bronchitis, backache, earache, rheumatism, thrombosis, sexual impotence, swelling, ophthalmological problems, sore throat, infection, thrombosis, swelling, injuries caused by spines of stingray, and pain relief in injuries caused by snake bites. Interestingly, caymans were also used as amulets to protect against snake bite or against evil eye [64, 65]. Likewise, the meat of armadillos (*Euphractus sexcintus* and *Dasypus novemcinctus*) was used as food, while their tail and skin were used for treating earache and asthma and as an amulet to protect against evil eye.

Another interface between the use of animals as food and medicine was expressed through the need, by those taking animal-based medicines, to control their diet—otherwise the medication would not work. Similar findings were described by Begossi [4] and Seixas and Begossi [78] who recorded the use of the word “carregado” to encompasses a set of supposed attributes of an animal (such as teeth, blood, aggressive behavior, “strong flesh,” and fattiness) and factors that could provoke an inflammation if the animal was eaten by a wounded or unhealthy person.

Although the main part used for alimentary purposes was the flesh, the eggs and viscera of some species were also used. Examples include the Amazon River turtle *Podocnemis expansa* (Schweigger, 1812), the Black Vulture *Coragyps atratus* (Bechstein, 1793), the smooth-billed ani *Crotophaga ani* (Linnaeus, 1758), the red-footed tortoise *Chelonoidis carbonaria* (Spix, 1824), the yellow-footed tortoise *Chelonoidis denticulata* (Linnaeus, 1766), and the domestic chicken *Gallus gallus* (Linnaeus, 1758).

The consumption of the meat of reptiles, mammals, birds, and fishes is often related to the purported medicinal or cultural benefits derived from the animal parts [62, 63, 74, 79–82], and this enduring relationship between food animals and medicinal therapy goes well beyond the understanding that adequate nutrition sustains a person's health. For instance, Werner [83] noted that much of the variation in the use or nonuse of lizards as food apparently stems from cultural beliefs concerning the medicinal or other benefits of their flesh. As an example of such cultural beliefs, in our study we found that some species used as food (e.g., *Crassostrea rhizophorae*, *Anomalocardia brasiliana*, and *Eunectes murinus*) were also considered to be aphrodisiacs.

Of the animals used as remedies and food, 52 (33.1%) are under some form of legal protection, a result that clearly indicates the need for bringing all relevant stakeholders together to develop strategies that can more effectively deal with the issues related to the harvesting of wildlife for alimentary and/or medicinal purposes in Brazil. As discussed by Alves and Rosa [1], sustainability of harvesting of medicinal animals is challenged by many factors, from both social and ecological perspectives, and it is important to respect differing views of the value of wildlife, while, at the same time, conserving biodiversity.

Connections between traditional medicine, biodiversity, and human health have recently been addressed by different authors [84–88] and have drawn attention to the fact that biodiversity loss can have indirect and direct effects on human well-being as well. The reliance on traditional uses of animals as food and as medicine by communities around the world highlights the need for further interdisciplinary research in ethnozoology which can be used in strategies to conserve biodiversity.

## Figures and Tables

**Table 1:** Animal species used as food and medicine in Brazil and conditions to which remedies are prescribed.

Taxonomic category/species	Conditions to which remedies are prescribed
Molluscs	
*Phacoides pectinata* (Gmelin, 1791)	Sexual impotence
*Mytella charruana* (Orbigny, 1842)	Ophthalmological problems
*Mytella guyanensis *(Lamarck, 1819)	Weakness
*Crassostrea rhizophorae* (Guilding, 1828)	Osteoporosis, pneumonia, stomach ache, cancer, flu, weakness, pain relief in injuries caused by the dorsal fin spine of a species of catfish, anemia, and tuberculosis
*Anomalocardia brasiliana* (Gmelin, 1791)	Asthma, flu, stomach and ache

Crustaceans	
*Cardisoma guanhumi* (Latreille, 1825)^NE/IN^	Asthma, bronchitis, wounds, and boils
*Goniopsis cruentata* (Latreille, 1802)	Epilepsy, venereal disease
*Ucides cordatus *(Linnaeus, 1763)^DD/IN^	Hemorrhage in women, incontinence, osteoporosis, cough, asthma, tuberculosis, womb disorders, arthrosis, and bronchitis
*Macrobrachium carcinus* (Linnaeus, 1758)	Amnesia
*Macrobrachium acanthurus* (Wiegmann, 1836)	Irritation when milk teeth are erupting
*Macrobrachium borellii* (Nobili, 1896)	Irritation when milk teeth are erupting
*Xiphopenaeus schmitti* (Burkenroad, 1936)	Irritation when milk teeth are erupting, skin spots
*Xiphopenaeus kroyeri* (Heller, 1862)	Irritation when milk teeth are erupting, skin spots
*Aratus pisonii *(H. Milne Edwards, 1837)	Epilepsy, to alleviate the symptoms of intoxication with poison of *Colomesus psittacus *(a species of pufferfish)

Fishes	
*Trachelyopterus galeatus* (Linnaeus, 1766)	Umbilical hernia, asthma, and sexual impotence
*Leporinus friderici* (Bloch, 1794)	Earache
*Schizodon knerii *(Steindachner, 1875)	Leucoma, edema
*Bagre bagre* (Linnaeus, 1766)	Injuries caused by itself
*Genidens barbus* (Lacepède, 1803)	Pain relief caused in injuries by the species' sting
*Genidens genidens *(Cuvier, 1829)	Injuries caused by itself
*Sciadeichthys luniscutis* (Valenciennes, 1837)	Pain relief caused in injuries by the species' sting
*Aspredo aspredo *(Linnaeus, 1758)	Asthma
*Aspredinichthys tibicen* (Valenciennes, 1840)	Asthma
*Balistes capriscus* (Gronow, 1854)^DD/IN^	Bronchitis
*Balistes vetula *(Linnaeus, 1758)^VU^	Stroke, asthma, thrombosis, earache, pain relief caused in injuries by the species' sting, hemorrhage, ascites, schistosomiasis, appendicitis, menstrual cramps, and gastritis
*Thalassophryne nattereri* (Steindachner, 1876)	Pain relief caused in injuries by the species' sting
*Callichthys callichthys* (Linnaeus, 1758)	Asthma, umbilical hernia
*Carcharhinus limbatus *(Müller and Henle, 1839)^LC^	Osteoporosis
*Carcharhinus porosus* (Ranzani, 1840)	Asthma, rheumatism, wounds, inflammations, osteoporosis, and anemia
*Galeocerdo cuvier *(Péron and Lesueur, 1822)^LC^	Osteoporosis
*Rhizoprionodon lalandii* (Müller and Henle, 1839)	Rheumatism
*Rhizoprionodon porosus* (Poey, 1861)	Rheumatism
*Sphyrna lewini *(Griffith and Smith, 1834)^LC/IN^	Asthma, wounds, rheumatism, and inflammation
*Centropomus parallelus* (Poey, 1860)	Nephritis
*Centropomus undecimalis* (Bloch, 1792)	Edema in the legs
*Astyanax bimaculatus* (Linnaeus, 1758)	Alcoholism, leishmaniasis, skin burns, wounds, and rheumatism
*Brycon nattereri* (Günther, 1864)	Flu
*Colossoma macropomum* (Cuvier, 1818)^DD/IN^	Paralysi of arms and legs
*Hydrolycus scomberoides* (Cuvier, 1816)	Earache
*Opisthonema oglinum* (Lesueur, 1818)	Alcoholism
*Dasyatis guttata *(Bloch and Schneider, 1801)	Asthma, pain relief caused in injuries by the species' sting, and burns
*Dasyatis marianae *(Gomes, Rosa, and Gadig, 2000)	Asthma, pain relief caused in injuries by the species' sting, and burns
*Franciscodoras marmoratus* (Reinhardt, 1874)	Injuries caused by itself
*Lithodoras dorsalis* (Valenciennes, 1840)	Swelling
*Megalodoras uranoscopus* (Eigenmann and Eigenmann, 1888)	Rheumatism
*Platydoras costatus* (Linnaeus, 1758)	Rheumatism
*Pterodoras granulosus* (Valenciennes, 1821)	Rheumatism
*Oxydoras niger* (Valenciennes, 1821)	Rheumatism
*Echeneis naucrates * Linnaeus, 1758	Asthma, bronchitis
*Erythrinus erythrinus* (Bloch and Schneider, 1801)	Asthma
*Hoplias malabaricus* (Bloch, 1794)	Ophthalmological problems, rheumatism, cataracts, wounds, snake bite, conjunctivitis, stroke, thrombosis, asthma, toothache, fever, earache, diarrhea, deafness, boils, bleedings, alcoholism, tetanus, sore throat, itching, sprains, and leucoma
*Gadus morhua* (Linnaeus, 1758)^VU^	Boils
*Ginglymostoma cirratum* (Bonnaterre, 1788)^DD^	Rheumatism
*Pimelodella brasiliensis* (Steindachner, 1876)	Injuries caused by that fish species
*Holocentrus adscensionis* (Osbeck, 1765)	Wounds
*Megalops atlanticus* (Valenciennes, 1847)	Stroke, headache, asthma, shortness of breath, thrombosis, chest pain, and injuries caused by bang
*Gymnothorax funebris* (Ranzani, 1840)	Bleeding (wounds)
*Gymnothorax moringa* (Cuvier, 1829)	Bleeding (wounds)
*Gymnothorax vicinus* (Castelnau, 1855)	Bleeding (wounds)
*Aetobatus narinari* (Euphrasen, 1790)^LC^	Asthma, pain relief caused in injuries by the species' sting, burns, and hemorrhage
*Narcine brasiliensis* (Olfers, 1831)	Toothache
*Arapaima gigas* (Schinz, 1822)^DD/II/IN^	Asthma, pneumonia
*Phractocephalus hemioliopterus* (Bloch and Schneider, 1801)	Asthma, wounds, hernia, burns in the skin, rheumatism, flu, and cough
*Pseudoplatystoma corruscans* (Spix and Agassiz, 1829)	Flu
*Pseudoplatystoma fasciatum* (Lunnaeus, 1776)	Cold
*Sorubimichthys planiceps* (Spix and Agassiz, 1829)	Leishmaniasis, tuberculosis
*Zungaro zungaro* (Humboldt, 1821)^DD/IN^	Asthma, toothache, earache, wounds, athlete's foot, burns in the skin, rheumatism, and flu
*Paratrygon aiereba* (Müller and Henle, 1841)	Asthma, hernia, flu, pneumonia, cough, earache, and burns
*Potamotrygon hystrix* (Müller and Henle, 1834)	Asthma, hernia, flu, pneumonia, cough, earache, and burns
*Potamotrygon motoro* (Müller and Henle, 1841)	Asthma, hernia, flu, pneumonia, cough, earache, and burns
*Potamotrygon orbignyi* (Castelnau, 1855)	Pain relief caused in injuries by that species' sting
*Plesiotrygon iwamae *(Rosa, Castello, and Thorson, 1987)	Pain relief caused in injuries by the species' sting, wounds, and cracks in the sole of the feet
*Pristis pectinata* (Latham, 1794)^CR^	Asthma, rheumatism, and arthritis
*Pristis perotteti *(Müller and Henle, 1841)^CR^	Asthma, rheumatism, and arthritis
*Prochilodus argenteus* (Spix and Agassiz, 1829)	To avoid swelling of the breast feeding, mycosis
*Prochilodus nigricans* (Spix and Agassiz, 1829)	Chilblain, skin burns, wounds, rheumatism, and eye pains
*Atlantoraja cyclophora* (Regan, 1903)^VU^	Hemorrhage after delivery
*Mylossoma duriventre* (Cuvier, 1818)	Venereal disease
*Serrasalmus brandtii* (Lütken, 1875)	Inflammations, sexual impotence
*Cynoscion acoupa* (Lacepède, 1801)	Renal failure
*Cynoscion leiarchus* (Cuvier, 1830)	Renal failure
*Micropogonias furnieri* (Desmarest, 1823)	Pain relief caused in injuries by the species' sting, cough, asthma, and bronchitis
*Pachyurus francisci* (Cuvier, 1830)	Asthma, urinary incontinence, and backache
*Plagioscion surinamensis* (Bleeker, 1873)	Urinary disorders, hemorrhage, and snake bites
*Plagioscion squamosissimus* (Heckel, 1840)	Urinary disorders, hemorrhage, and snake bites
*Calamus penna *(Valenciennes, 1830)	Asthma
*Synbranchus marmoratus* (Bloch, 1795)	Bronchitis
*Colomesus psittacus *(Bloch and Schneider, 1801)	Breast cancer, backache, and warts
*Sphoeroides testudineus* (Linnaeus, 1758)	Rheumatism
*Trichiurus lepturus* (Linnaeus, 1758)	Asthma
*Urotrygon microphthalmum* (Delsman, 1941)	Asthma, pain relief caused in injuries by the species' sting, and burns

Reptiles	
*Iguana iguana *(Linnaeus, 1758)^DD/II^	Earache, erysipelas, asthma, rheumatism, edema, abscesses, joint pain, wounds, acne, athlete's foot, sore throat, swelling, burn, tumor, to suck a splinter out of skin or flesh, boil, injuries caused by the spines of the “arraia” and others fishes, inflammation, and hernia
*Tupinambis merianae* (Duméril and Bibron, 1839)^DD/II^	Earache, deafness, rheumatism, erysipelas, skin thorns and wounds, respiratory diseases, sore throat, snake bite, asthma, tumor, swelling, infection, and bronchitis
*Tupinambis teguixin* (Linnaeus 1758)^DD/II^	Sexual impotence, rheumatism, erysipelas, dermatitis, snake bites, asthma, tetanus, earache, thrombosis, wounds, paronychia, swelling, herpes zoster, irritation when milk teeth are erupting, jaundice, inflammation, tumor, sore throat, infection, bronchitis, injuries caused by the spines of the “arraia,” pain relief in injuries caused by snake bites, toothache, suck a splinter out of skin or fresh, headache, cough, stroke, and coarse throat
*Boa constrictor *(Linnaeus, 1758)^DD/II^	Rheumatism, lung disease, thrombosis, boils, tuberculosis, stomach ache, edema, snake bite, cancer, ache, swelling, to prevent abort, pain in the body, inflammation, athlete's foot, calluses, tumors, cracks in the sole of the feet, goiter, sore throat, arthrosis, insect sting, dog bite, erysipelas, thrombosis, asthma, neck strain, and strain muscle
*Eunectes murinus *(Linnaeus, 1758)^DD/II^	Wounds, skin problems, bruises, sprains, arthrosis, rheumatism, boils, sexual impotence, headache, sore throat, thrombosis, swelling, tumour, asthma, muscle strain, numbness, syphilis, to reduce pain, and luxation
*Caretta caretta *(Linnaeus, 1758)^VU/I^	Injuries caused by bang, toothache, diabetes, headache, backache, wounds, cough, bronchitis, asthma, thrombosis, rheumatism, stroke, hoarseness, flu, backache, earache, sore throat, and swelling
*Chelonia mydas *(Linnaeus, 1758)^VU/I^	Injuries caused by bang, toothache, diabetes, headache, backache, wounds, cough, bronchitis, asthma, flu, thrombosis, rheumatism, toothache, stroke, hoarseness, earache, sore throat, swelling, whooping cough, arthritis, erysipelas, boil, wounds, arthrosis, and inflammation
*Eretmochelys imbricata* (Linnaeus, 1766)^EN/I^	Injuries caused by bang, toothache, diabetes, headache, backache, wounds, cough, bronchitis, asthma, thrombosis, stroke, hoarseness, flu, rheumatism, earache, sore throat, and swelling
*Lepidochelys olivacea *(Eschscholtz, 1829)^EM/I^	Injuries caused by bang, toothache, diabetes, headache, backache, wounds, cough, flu, bronchitis, asthma, thrombosis, rheumatism, stroke, and hoarseness
*Dermochelys coriacea* (Vandelli, 1761)^CR/I^	Rheumatism, earache, sore throat, and swelling
*Rhinoclemmys punctularia* (Daudin, 1802)	Wounds, tumor, erysipelas, earache, and rheumatism
*Podocnemis expansa* (Schweiger, 1812)^LC/II^	Inflammation, acne, tumor, boil, rheumatism, pterygium, skin spots, backache, earache, arthrosis, arthritis, swelling, and wrinkle
*Podocnemis unifilis* (Troschel, 1848)^VU/II^	Wounds, tumor, erysipelas, earache, and rheumatism
*Podocnemis sextuberculata* (Cornalia, 1849)	Blackhead; acne
*Peltocephalus dumerilianus * (Schweigger, 1812)	Blackhead; acne
*Caiman crocodilus* (Linnaeus, 1758)^LC/II^	Asthma, stroke, bronchitis, backache, earache, rheumatism, thrombosis, sexual impotence, snake bites (antidote), evil eye, irritation when milk teeth are erupting, discharge, swelling, scratch, athlete's foot, ophthalmological problems, asthma, sore throat, amulet used as a protection against snake bite, rheumatism, hernia, prostate problems, infection, and thrombosis
*Caiman latirostris* (Daudin, 1801)^LC/II^	Asthma, sore throat, amulet used as a protection against snake bite, rheumatism, irritation when milk teeth are erupting, hernia, and prostate problems
*Melanosuchus niger* (Spix, 1825)^LC/II^	Thrombosis, infection, swelling, asthma, amulet used as a protection against snake bite, injuries caused by spines of the “arraia,” and pain relief in injuries caused by snake bites
*Paleosuchus palpebrosus* (Cuvier, 1807)^LC/II^	Snake bite, asthma, stroke, rheumatism, thrombosis, backache, sexual impotence, edema, mycosis, evil eye, irritation when milk teeth are erupting, snake bite (antidote), discharge, sore throat, amulet used as a protection against snake bite, hernia, and prostate problems
*Paleosuchus trigonatus* (Schneider, 1801)^DD/II^	Rheumatism

Birds	
*Anser anser* (Linnaeus, 1758)	Laryngitis, pharyngitis, and tonsillitis
*Ardea cocoi *(Linnaeus, 1766)	Swelling, inflammation, injuries caused by the spines of the “arraia” and others fishes, asthma, boil, tumor, inflammation, rheumatism, and earache
*Penelope jacucaca* (Spix, 1825)^VU^	Insomnia
*Ciconia maguari* (Gmelin, 1789)	Injuries caused by the spines of the “arraia” and others fishes, and thrombosis
*Leptotila rufaxilla* (Richard and Bernard, 1792)	Thrombosis
*Columba livia* (Gmelin, 1789)	Asthma, laryngitis, pharyngitis, and tonsillitis
*Meleagris gallopavo* (Linnaeus, 1758)	Asthma
*Rhea americana* (Linnaeus, 1758)^LC/II^	General aches, rheumatism, thrombosis, and strokes
*Crypturellus noctivagus* (Wied, 1820)^VU^	Thrombosis, stroke
*Nothura boraquira* (Spix, 1825)	Thrombosis, stroke
*Rhynchotus rufescens* (Temminck, 1815)	Snake bite, thrombosis, and snake bites (antidote)
Mammals	
*Agouti paca *(Linnaeus, 1766)^LC/III^	Wound in the breast caused by suckling, ophthalmological problems, stomach disorders, pterygium, to suck a splinter out of skin or flesh, injuries caused by the spines of “arraia,” and control cholesterol level
*Bubalus bubalis* (Linnaeus, 1758)	Rheumatism, osteoporosis, and thrombosis
*Capra hircus *(Linnaeus, 1758)	Evil eye, snake bite, and muscle strain
*Bradypus variegatus* (Shinz, 1825)	Thrombosis
*Bradypus tridactylus* (Linnaeus, 1758)	Thrombosis, insects bite, and scorpions bite
*Cavia aperea* (Erxleben, 1777)	Inflammation
*Kerodon rupestris* (Wied-Neuwied, 1820)	Constipation
*Alouatta belzebul* (Linnaeus, 1766)^CR^	Whooping cough, sore throat, and asthma
*Alouatta nigerrima *(Lönnberg, 1941)	Whooping cough, inflammation
*Alouatta seniculus *(Linnaeus, 1766)^LC/II^	Whooping cough, inflammation, and to accelerate parturition
*Cebus apella* (Linnaeus, 1758)^LC/II^	Insect sting
*Blastocerus dichotomus* (Illiger, 1815)^VU/I^	Diarrhea, vomit
*Mazama americana* (Erxleben, 1777)^DD/III^	Stroke
*Mazama simplicicornis* (Illinger, 1811)	Diarrhea, verminosis, and evil eye
*Mazama *cf. *gouazoubira* (G. Fischer, 1814)	Asthma, edema, rheumatism, snake bite, thrombosis, to assist children who take longer than usual to start walking, toothache, wounds, and sprains
*Ozotocerus bezoarticus* (Linnaeus, 1758)	Diarrhea, verminosis, and evil eye
*Dasypus novemcinctus* (Linnaeus, 1758)	Thrombosis, insects bite, scorpions bite, edema, asthma, deafness, earache, and evil eye
*Euphractus sexcinctus* (Linnaeus, 1758)	Wounds, earache, evil eye, asthma, sore throat, pneumonia, sinusitis, deafness, and coarse throat
*Tolypeutes tricinctus* (Linnaeus, 1758)^VU^	Thrombosis, rheumatism
*Dasyprocta prymnolopha* (Wagler, 1831)	Asthma, thrombosis
*Sotalia fluviatilis *(Gervais and Deville, 1853)^DD/I^	Asthma, headache, rheumatism, hernia, womb disorders, sore throat, injuries caused by the spines of the “arraia,” swelling, hemorrhoids inflammation, wounds, earache, erysipelas, athlete's foot, tumor, and cancer
*Sotalia guianensis* (P. J. Van Bénéden, 1864)	Asthma, headache, rheumatism, hernia, womb disorders, sore throat, injuries caused by the spines of the “arraia,” swelling, hemorrhoids inflammation, wounds, earache, erysipelas, athlete's foot, tumor, and cancer
*Coendou prehensilis* (Linnaeus, 1758)	Bronchitis, thrombosis, epilepsy, stroke, abscesses, conjunctivitis, and asthma
*Hydrochaeris hydrochaeris* (Linnaeus, 1766)	Thrombosis, conjunctivitis, venereal disease, rheumatism, earache, strengthen bones, liver pain, bronchitis, asthma, wounds, erysipelas, and cough
*Inia geoffrensis *(Blainville, 1817)^VU/II^	Asthma, headache, rheumatism, hernia, womb disorders, sore throat, injuries caused by the spines of the “arraia,” swelling, hemorrhoids inflammation, wounds, earache, erysipelas, athlete's foot, tumor, and cancer
*Sylvilagus brasiliensis* (Linnaeus, 1758)	Thrombosis, conjunctivitis, boils, and burns
*Conepatus semistriatus* (Boddaert, 1785)	Rheumatism
*Lontra longicaudis* (Olfers, 1818)^DD/I^	Thrombosis
*Myrmecophaga tridactyla* (Linnaeus, 1758)^VU/II^	Thrombosis, stroke
*Myrmecophaga tetradactyla* (Linnaeus, 1758)	Edema, thrombosis
*Tapirus terrestris *(Linnaeus, 1758)^VU/II^	Rheumatism, arthrosis, osteoporosis, bursitis, muscular pain, asthma, and tonsillitis
*Pecari tajacu* (Linnaeus 1758)^LC/II/III^	Thrombosis, bronchitis, and stroke
*Tayassu pecari* (Link, 1795)^LC/II^	Thrombosis, stroke
*Trichechus inunguis* (Natterer, 1883)^VU/I^	Sprains, vaginal discharge, injuries caused by bang, burns, asthma, menstrual cramps, rheumatism, sore throat, wounds, muscle strain, suck a splinter out of skin or fresh, tumor, backache, hernia, arthrosis, luxation, menstrual cramps, and insects bite
*Trichechus manatus *(Linnaeus, 1758)^CR/I^	Sprains, vaginal discharge, injuries caused by bang, burns, asthma, menstrual cramps, rheumatism, sore throat, wounds, muscle strain, suck a splinter out of skin or fresh, tumor, backache hernia, arthrosis, luxation, menstrual cramps, and insects bite

Categories of IUCN Red List: CR: critically endangered, EN: endangered, VU: vulnerable, LC: least concern, DD: data deficient, and NE: not evaluated. Cites appendices (I, II, and III); IN: Anexo 2–Instrução Normativa n. 5/2004/MMA.
